# Supplementary material for: The Synergy of Chicken Anemia Virus and Gyrovirus Homsa 1 in Chickens
Source: Viruses. 2023 Feb 13;15(2):515. doi: 10.3390/v15020515 (PMC9964263; doi:10.3390/v15020515)
Supplement: Supplementary file 1 [file viruses-15-00515-s001.zip › Supplementary Figure S1.pdf]

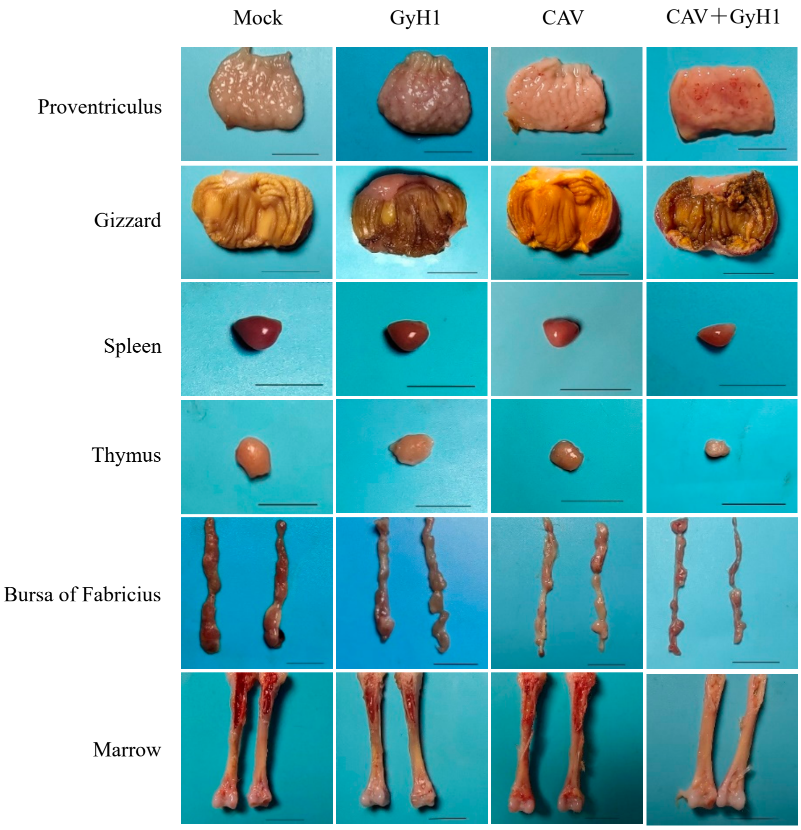


Supplementary Figure S1. Pathologically anatomic lesions at 14dpi, ruler = 1cm. Glandular gastric mucosa hemorrhage. Myogastric ulcer erosion. Atrophy of the spleen, bursa of Fabricus, and thymus. The bone marrow is filled with fat and turns yellow.
